# What are people’s attitudes toward medical insurance fraud whistleblowers? a study from China

**DOI:** 10.1186/s12889-023-17606-3

**Published:** 2024-01-03

**Authors:** Fangting Chen, Fangmin Deng, Jingran He, Jinpeng Xu, Jian Liu, Qi Shi, Hongyu Zhang, Ting Zhang, Qunhong Wu, Lijun Gao, Guomei Tian, Zheng Kang

**Affiliations:** 1 School of Health Management, Harbin Medical University, Harbin, Heilongjiang China; 2 Department of Nuclear Medicine, The Fourth Affiliated Hospital of Harbin Medical University, Harbin, Heilongjiang China

**Keywords:** Medical Insurance Fraud, Whistleblower, Attitude, China

## Abstract

**Purpose:**

Medical insurance fraud has caused huge losses to countries around the world, and public reporting has become an important means to combat medical insurance fraud. The attitude of medical insurance fraud whistleblowers affects people’s reporting behavior, and understanding people’s attitude toward medical insurance fraud whistleblowers provides a basis for further improving the system and policy of public participation in medical insurance fund supervision.

**Methods:**

We adopted the questionnaire method to conduct a national cross-sectional survey of the Chinese public and analyzed the data using Chi-square tests, Fisher’s exact tests, and binary logistic regression models.

**Results:**

A total of 837 respondents were included, and 81.8% of the population had a supportive attitude toward medical insurance fraud whistleblowers, with gender, whether they had used medical insurance reimbursement, and present life satisfaction being statistically significant (*P* < 0.05).

**Conclusion:**

The public is generally supportive of medical insurance fraud whistleblowers, and women, those who have used medical insurance for reimbursement, and those who are satisfied with their lives are more likely to be supportive of medical insurance fraud whistleblowers.

Medical insurance fraud is primarily an act of insurance fraud committed by an individual or organization by means of intentional deception, falsehood, or concealment, in order to obtain some benefit from the violation of the law [1]. Medical insurance fraud has caused huge financial losses to medical insurance funds around the world [2, 3]. In the United States, medical insurance fraud results in tens of billions of dollars in losses each year [4, 5]. In India, medical insurance fraud accounts for about 15% of total claims [6], and in China, medical insurance fraud accounts for about 7–8% of national healthcare costs [7]. Therefore, there is an urgent need to combat medical insurance fraud.

As the number of medical insurance fraud incidents continues to increase [8, 9], fraudulent methods continue to shift from the explicit to the implicit, and fraudulent behavior continues to evolve and remain hidden [10], posing new challenges to the supervision of medical insurance funds. Whistleblowing is the most common and effective method of detecting fraud [11]. Whistleblowing is also widely recognized internationally as the act of bringing to the attention of an organization’s current or former employees the unlawful, illegal or unethical conduct of their employer to the attention of an organization or individual who can take action against them [12]. For example, many of the failures of the NHS in the UK have come to light through the whistleblower’s whistleblowing behavior [13]. According to statistics, 90% of medical insurance fraud revelations in the United States between 1996 and 2005 were initiated by whistleblowers [14].

Medical insurance fraud whistleblowers are all individuals or organizations who report to individuals or organizations capable of committing effective acts of intentional misuse or fraudulent use of the medical insurance fund by using deceptive means such as fictitious or exaggerated medical conditions, concealment of facts, and other deceptive means [1, 12]. In China, when people find medical insurance fraud, they can report it to the government, media and relevant medical insurance departments by phone, letter, email, message on the government’s official website, so on. There are two options for anonymous and real-name reporting, and the whistleblower is free to choose which way to report, and upon receipt of a report, the relevant authorities, once verified, will reward the informant and publicize the typical incident on the official website in order to encourage and alert the public, it can be seen that the role of the medical insurance fraud whistleblower can not be ignored. Whistleblowers can be seen playing an important role in exposing medical insurance fraud [15], but people have different perceptions of whistleblowers. On the one hand, whistleblowers are seen as a troublemaker [16, 17] while on the other hand, they are seen as heroes and “pro-social behavior” that supports the public interest [18–20]. People’s attitudes toward whistleblowers reflect the cultural environment and social support for whistleblowing behavior and are important environmental factors in the creation of whistleblowing behavior. Therefore, it is essential to understand people’s attitudes toward medical insurance fraud whistleblowers.

People report fraud more for the sake of maintaining public interest or public order than for seeking personal benefits [13, 15, 21]. People’s attitudes toward whistleblowing and the whistleblower largely influence their whistleblowing behavior when faced with other scenarios that undermine the public interest or public order. To our knowledge, most studies have addressed corporate fraud whistleblowers from the perspectives of the legal system of whistleblowing [22], the whistleblower reward system [23], and the whistleblower protection system [24], and no studies have been found on the attitudinal aspects of people who are whistleblowers of medical insurance fraud. Therefore, this paper attempts to analyze people’s attitudes toward medical insurance fraud whistleblowers, further enriching the theoretical study of public participation in the management and governance of public affairs, and providing a reference for advancing anti-fraud research, as well as analyzing people’s attitudes toward whistleblowers in other public areas such as environmental pollution and food safety.

## Materials and methods

### Study design and participants

This study used the questionnaire method, which was sent to respondents through a widely accepted online questionnaire platform in China. The details are as follows:

First, before conducting a formal survey on the questionnaire, we pre-surveyed using convenience sampling and repeatedly revised the questionnaire based on the feedback to finally form the formal questionnaire. Second, we measured the sample size by calculating the formula as a way to ensure the feasibility of the sample size. The formula for sample size is as follows.

$$n = \frac{{{z^2} * P * (1 - P)}}{{{d^2}}} $$

Where z is the confidence level. *z* is usually 1.96, which is the 95% confidence level we chose; *P* is the percentage of a feature in the target population, which is set to 0.5 since there is no a priori data; and *d* is the acceptable level of precision/accuracy, which is usually set to 0.04. According to the formula, 601 samples should be analyzed in this study.

Third, the official questionnaire was used to conduct a national cross-sectional study using an online platform from February 19 to September 13, 2022. As the researchers were from different provinces, they first distributed the questionnaire links in their province and then proceeded with data collection in a snowball fashion. The division of China into East, Central and West according to the China Health Statistics Yearbook. The representatives of the eastern region were selected from Jiangsu and Liaoning Provinces. The representatives of the central region were chosen from Heilongjiang and Anhui Provinces. The western region were represented by Guizhou and Shaanxi Provinces. Fourth, survey respondents were selected from the Chinese public aged 15 and above. Finally, the validity of the questionnaire was judged based on whether each IP address was limited to one response, whether the response time was more than 10 min, whether the two quality control questions were correctly completed, and whether the response options were regular. A total of 837 valid questionnaires were finally collected after the screening. All respondents volunteered to participate in the study.

### Variables

#### Dependent variable

**Attitude**, which indicates the respondent’s attitude toward medical insurance fraud whistleblowers. Attitude is a binary variable: not supportive or supportive.

This variable was defined based on respondents’ responses to the following question in the questionnaire: “If someone reported medical insurance fraud, what would be your attitude toward the whistleblower?” The responses to this question were “not at all supportive, relatively unsupportive, so-so, relatively supportive, and very supportive”. We classify “not at all supportive, relatively unsupportive, and so-so” as not supportive attitudes, and “relatively supportive and very supportive” as supportive attitudes.

#### Independent variables

All independent variables were categorical variables:

They include gender, age, education level, marital status, occupation type, annual household income, whether they were enrolled in medical insurance, insurance location, whether they had used medical insurance reimbursement, whether the family member has a chronic disease, whether the family member has had a serious illness, whether the family member has a medical worker, whether the family member has a medical insurance worker, and satisfaction with present life.

### Statistical analysis

To explore the differences between people’s attitudes toward medical insurance fraud whistleblowers and the factors influencing them, the following methods were used:

First, all data in this study were statistically analyzed using SPSS 26.0 with a two-sided test level of α = 0.05. Second, frequencies and percentages were used to characterize the public and supportive attitudes under both attitudes. Third, Chi-square tests and Fisher’s exact tests were used to determine the differences and significance between public attitudes toward medical insurance fraud whistleblowers and different categories of independent variables. Finally, binary logistic regression was used to determine the factors that may influence public attitudes toward medical insurance fraud whistleblowers under two attitudes.

## Results

### General characteristics of participants

Among the 837 respondents, 324 were male (38.7%) and 513 were female (61.3%). 76.5% of the respondents were young people, 52.1% were bachelor’s or college, more than half were unmarried, 30.1% were engaged in other occupations, and 51.3% of households have annual incomes greater than 50,000CNY and less than or equal to 150,000CNY. In addition, among the respondents in this study, 88.8% indicated that they were enrolled in medical insurance, 63.6% of participants were from the Eastern region, 51.4% had not used medical insurance reimbursement, 79.9% indicated that there were no medical workers among family members, 91.2% indicated that there were no medical insurance workers among family members, and 56.8% were satisfied with their present life (Table 1).

**Table 1:** A Single-factor Analysis of People’s Attitudes toward Medical Insurance Fraud Whistleblowers [N = 837]

Variables	Categories	Respondents N (%)	Not supportive n(%)	Supportive n(%)	X^2^	*P-*value
Gender	Male	324(38.7)	71(46.7)	253(36.9)	5.011	0.025
	Female	513(61.3)	81(53.3)	432(63.1)		
Age	15–17	11(1.3)	1(0.7)	10(1.5)	4.013	0.260
	18–44	640(76.5)	111(73.0)	529(77.2)		
	45–59	167(20.0)	38(25.0)	129(18.8)		
	60 and above	19(2.3)	2(1.3)	17(2.5)		
Education level	Middle School and below	139(16.6)	36(23.7)	103(15.0)	9.944	0.019
	High School or technical secondary school	108(12.9)	24(15.8)	84(12.3)		
	Bachelor’s or college	436(52.1)	65(42.8)	371(54.2)		
	Master and above	154(18.4)	27(17.8)	127(18.5)		
Marital status	Unmarried	444(53.0)	80(52.6)	364(53.1)	4.845	0.164
	Married	366(43.7)	63(41.4)	303(44.2)		
	Divorced	22(2.6)	7(4.6)	15(2.2)		
	Widowed	5(0.6)	2(1.3)	3(0.4)		
Occupation type	Government agencies	145(17.3)	21(13.8)	124(18.1)	10.804	0.029
	Enterprise workers	173(20.7)	28(18.4)	145(21.2)		
	Other	252(30.1)	59(38.8)	193(28.2)		
	Students	227(27.1)	33(21.7)	194(28.3)		
	Retirees	40(4.8)	11(7.2)	29(4.2)		
Annual household income	≤ 50,000 CNY	244(29.2)	55(36.2)	189(27.6)	6.175	0.100
	> 50,000 ≤ 150,000	429(51.3)	76(50.0)	353(51.5)		
	> 150,000 ≤ 300,000	135(16.1)	18(11.8)	117(17.1)		
	> 300,000 CNY	29(3.5)	3(2.0)	26(3.8)		
Medical insurance	Uninsured	94(11.2)	18(11.8)	76(11.1)	0.070	0.792
	Insured	743(88.8)	134(88.2)	609(46.0)		
Insurance Location	Uninsured	94(11.2)	18(11.8)	76(11.1)	3.760	0.289
	Eastern region	532(63.6)	105(69.1)	427(62.3)		
	Central region	84(10.0)	11(7.2)	73(10.7)		
	Western region	127(15.2)	18(11.8)	109(15.9)		
Whether you have used medical insurance reimbursement	Uninsured	94(11.2)	18(11.8)	76(11.1)	8.936	0.011
	No	430(51.4)	93(61.2)	337(49.2)		
	Yes	313(37.4)	41(27.0)	272(39.7)		
Whether there is a chronic disease in the family	No	541(64.6)	104(68.4)	437(63.8)	1.164	0.281
	Yes	296(35.4)	48(31.6)	248(36.2)		
Whether the family member has suffered from a serious illness	No	641(76.6)	126(82.9)	515(75.2)	4.126	0.042
	Yes	196(23.4)	26(17.1)	170(24.8)		
Whether there is a medical worker in the family members	No	669(79.9)	118(77.6)	551(80.4)	0.611	0.435
	Yes	168(20.1)	34(22.4)	134(19.6)		
Whether there are medical insurance workers in the family members	No	763(91.2)	134(88.2)	629(91.8)	2.075	0.150
	Yes	74(8.8)	18(11.8)	56(8.2)		
Satisfaction with present life	Unsatisfied	72(8.6)	15(9.9)	57(8.3)	13.961	0.001
	So-so	290(34.6)	71(46.7)	219(32.0)		
	Satisfied	475(56.8)	66(43.4)	409(59.7)		

### Single-factor analysis

Table 1 shows the results of descriptive and single-factor analysis of public attitudes toward medical insurance fraud whistleblowers. Among the respondents, 685 people (81.8%) had a supportive attitude toward medical insurance fraud whistleblowers, and 152 people (18.2%) had an unsupportive attitude, and the respondents’ attitudes showed that people had an overall supportive attitude toward medical insurance fraud whistleblowers.

More specifically, the survey of attitudes toward medical insurance fraud whistleblowers by age, marital status, annual household income, whether or not they were enrolled in medical insurance, insurance location, whether or not they had family members with chronic diseases, and whether or not they had medical and medical insurance workers among their family members showed no statistically significant differences (*P* > 0.05). There were statistically significant differences in the attitudes of medical insurance fraud reporters by gender, education level, occupation type, whether they had used medical insurance reimbursement, whether their family members had suffered from serious diseases, and their present life satisfaction (*P* < 0.05).

### Logistic regression analysis

A binary logistic regression analysis was conducted using people’s attitudes toward medical insurance fraud whistleblowers as the dependent variable (Not supportive = 0, supportive = 1). The results show that gender, whether they have used medical insurance reimbursement, and present life satisfaction are factors that influence people’s attitudes toward medical insurance fraud whistleblowers (*P* < 0.05). Females were more likely to be supportive of medical insurance fraud whistleblowers than males. Those who have not used medical insurance reimbursement are less likely to have a supportive attitude than those who have used medical insurance reimbursement. Those who are dissatisfied are less likely to be supportive of medical insurance fraud whistleblowers than those who are satisfied with their present lives. People with different education level, occupation type, and whether or not a family member had a serious illness did not influence the relationship between whistleblower attitudes (Table 2).

**Table 2:** Binary logistic regression analysis of attitudes affecting people’s attitudes toward medical insurance fraud whistleblowers

Variables	OR (95% *CI*)	*P-*value
Gender (Female)	0.551(0.374,0.813)	0.003
Age (60 and above)		0.249
15–17	0.339(0.020,5.792)	0.455
18–44	0.162(0.026,1.030)	0.054
45–59	0.188(0.032,1.092)	0.063
Education level (Master and above)		0.089
Middle School and below	0.704(0.289,1.716)	0.440
High School or technical secondary school	0.931(0.398,2.182)	0.870
Bachelor’s or college	1.520(0.837,2.759)	0.169
Marital status (Widowed)		0.067
Unmarried	4.293(0.554,33.253)	0.163
Married	7.083(0.974,51.507)	0.053
Divorced	3.925(0.433,35.605)	0.224
Occupation type (Retirees)		0.084
Government agencies	3.243(1.100,9.561)	0.033
Enterprise workers	2.615(0.959,7.132)	0.060
Other	2.078(0.811,5.322)	0.127
Students	4.525(1.503,13.624)	0.007
Annual household income (> 300,000 CNY)		0.573
≤ 50,000 CNY	0.489(0.133,1.795)	0.281
> 50,000 ≤ 150,000 CNY	0.523(0.148,1.847)	0.314
> 150,000 ≤ 300,000 CNY	0.694(0.184,2.620)	0.590
Medical insurance (Insured)	0.593(0.269,1.309)	0.196
Insurance Location (Western region)		0.126
Eastern region	0.630(0.350,1.133)	0.123
Central region	1.110(0.471,2.615)	0.812
Whether you have used medical insurance reimbursement (Yes)		0.038
No	0.633(0.411,0.975)	0.038
Whether there is a chronic disease in the family (Yes)	0.997(0.640,0.640)	0.990
Whether the family member has suffered from a serious illness (Yes)	0.642(0.382,1.080)	0.095
Whether there is a medical worker in the family members (Yes)	1.420(0.860,2.345)	0.170
Whether there are medical insurance workers in the family members (Yes)	1.539(0.824,2.875)	0.176
Satisfaction with present life (Satisfied)		0.011
Unsatisfied	0.601(0.309,1.169)	0.134
So-so	0.544(0.363,0.815)	0.003

### Stability testing

In order to test the robustness of the results of the regression analysis, this study used the alternative variable method for stability testing. Substitute whether you have a chronic illness and whether you have ever had a serious illness for whether you have a family member with a chronic illness and whether your family member has ever had a serious illness. Based on the regression results, it can be seen that gender, whether or not one has used medical insurance reimbursement, and life satisfaction still significantly affect public attitudes toward medical insurance fraud whistleblowers after replacing the variables (*P* < 0.05), and thus the regression results are robust (Table 3).

**Table 3:** Stability testing results

Variables	OR (95% *CI*)	*P-*value
Gender (Female)	0.519(0.351,0.768)	0.001
Age (60 and above)		0.337
15–17	0.473(0.028,7.981)	0.604
18–44	0.209(0.034,1.289)	0.092
45–59	0.230(0.041,1.295)	0.096
Education level (Master and above)		0.096
Middle School and below	0.753(0.311,1.824)	0.530
High School or technical secondary school	1.002(0.430,2.335)	0.996
Bachelor’s or college	1.563(0.865,2.823)	0.139
Marital status (Widowed)		0.076
Unmarried	3.714(0.486,28.413)	0.206
Married	6.087(0.847,43.738)	0.073
Divorced	3.130(0.346,28.283)	0.310
Occupation type(Retirees)		0.564
Government agencies	0.477(0.131,1.740)	0.262
Enterprise workers	0.496(0.141,1.747)	0.275
Other	0.657(0.174,2.475)	0.534
Students		0.045
Annual household income (> 300,000 CNY)	3.523(1.200,10.345)	0.022
≤ 50,000 CNY	2.795(1.033,7.564)	0.043
> 50,000 ≤ 150,000 CNY	2.154(0.846,5.484)	0.108
> 150,000 ≤ 300,000 CNY	5.077(1.697,15.190)	0.004
Medical insurance (Insured)	0.548(0.249,1.208)	0.136
Insurance Location (Western region)		0.130
Eastern region	0.637(0.354,1.146)	0.132
Central region	1.124(0.478,2.643)	0.789
Whether you have used medical insurance reimbursement (Yes)		0.043
No	0.639(0.414,0.986)	0.043
Do you have a chronic disease (Yes)	0.677(0.304,1.506)	0.339
Have you ever suffered from a serious illness (Yes)	0.622(0.222,1.744)	0.367
Whether there is a medical worker in the family members (Yes)	1.438(0.872,2.372)	0.155
Whether there are medical insurance workers in the family members (Yes)	1.555(0.834,2.900)	0.165
Satisfaction with present life(Satisfied)		0.008
Unsatisfied	0.602(0.311,1.167)	0.133
So-so	0.530(0.354,0.795)	0.002

## Discussion

In this study, we found that 81.8% of respondents indicated support for medical insurance fraud whistleblowers, and people were more likely to be supportive of medical insurance fraud whistleblowers.

Our study found differences in attitudes toward whistleblowers among people with different gender characteristics, with females being more likely to have a supportive attitude toward whistleblowers compared to males. Medical insurance fraud involves illegal and ethical behavior that endangers the public interest of society and greatly damages the quality of medical services for people [8]. Some studies suggest that females are more likely to be more active in promoting public service, have more equity and altruism, and have less tolerance for unethical behavior [25, 26]. Similarly, previous research has shown that females are more likely to report unethical behavior [27]. These findings are consistent with those of Brolo [28]and Goetz [29], who concluded that women were more likely to act as whistleblowers when confronted with immoral behavior. Therefore, this may be the reason why females are more supportive of medical insurance fraud whistleblowers than males.

Another important finding was that those who had used medical insurance reimbursement were more likely to have a supportive attitude than those who had not used medical insurance reimbursement. It is possible that those who have been reimbursed by medical insurance are more aware of the importance of medical insurance and are more interested in ensuring that these funds are not wasted or misused. A study suggest that consumers with more experience in consuming, enrolling in and settling insurance claims are less likely to tolerate insurance fraud around them [30]. These are likely the reasons why people who have used medical insurance reimbursement are more supportive of medical insurance fraud whistleblowers.

Our study also found that those who expressed satisfaction with their lives were more likely to be supportive of medical insurance fraud whistleblowers. Taylor [20] believed that the whistleblowers was engaging in pro-social behavior, while Cunningham [31] also believed that life satisfaction could promote pro-social behavior. In psychological research, emotions influence personal decisions and behaviors. People with high life satisfaction are more capable of positive emotional responses [32], and those with high life satisfaction tend to have higher health literacy [33], which means they have stronger health beliefs and safety consciousness [34], and will pay more attention to the safety and effectiveness of medical insurance funds. As a result, individuals with high levels of life satisfaction are more likely to take action to support medical insurance fraud whistleblowers in situations where they face significant damage from medical insurance fraud.

In conclusion, understanding the public’s attitude toward whistleblowers and encouraging a supportive attitude to promote whistleblowers’ disclosure behavior will have a significant contribution to strengthening the fight against medical insurance fraud, achieving sustainability of medical insurance funds and safeguarding public health equity. Therefore, the government should attach great importance to the contribution made by whistleblowers to whistleblowing, establish correct and reasonable related mechanisms, and give correct value guidance in order to improve the supportive attitude of the public towards whistleblowers, create a favorable social and cultural atmosphere, raise public awareness of participation, and jointly safeguard the interests of the public. In addition, the public should improve their medical insurance policy literacy and monitoring awareness to quickly identify fraudulent insurance practices and reduce the possibility of fraudulent use of medical insurance funds, which will have a positive effect on the effective use of medical insurance funds and the efficient development of the healthcare system, with a view to providing lessons for better public participation in the regulation of medical insurance funds and other public areas in the future.

### Advantages and limitations of the study

This study has several strengths. First, current research focuses more on whistleblower protection systems and less on public attitudes toward whistleblowers. Second, our study is a supportive attitude toward medical insurance fraud whistleblowers, a topic that, to our knowledge, has not yet been studied.

This study also has several limitations. First, the data were collected through a self-report questionnaire and may be affected by factors such as self-reporting errors. Second, the respondents in this study were all from China, which means that it is limited to reflecting the attitudes of the Chinese public toward medical insurance fraud whistleblowers and therefore cannot be analyzed and generalized to other countries. In future studies, we will continue to focus on people’s attitudes toward medical insurance fraud whistleblowers to create a more comprehensive analysis of whistleblower attitudes.

## Conclusions

This study provides insight into people’s attitudes toward medical insurance fraud whistleblowers, and the results show that people are generally supportive of whistleblowers. It was further found that people’s attitudes toward whistleblowers were strongly associated with gender, whether they had used medical insurance reimbursement, and life satisfaction. In addition, all stakeholders should be concerned about specific negative perceptions of whistleblowers in the medical insurance fraud field. If retained, these negative attitudes may influence their attitudes toward whistleblowers and thus become a barrier to people being afraid to report and unwilling to do so.

Accordingly, there is an urgent need to increase the publicity coverage of the dangers of medical insurance fraud and the contributions made by whistleblowers, and to identify interventions, especially practical legal protection, and anti-stigma interventions, which are important for combating medical insurance fraud, maintaining the safety of medical insurance funds, and improving the policies for the regulation of medical insurance funds.

## Data Availability

Our funded project belongs to the National Natural Science Foundation of China and has not yet been completed, so we are not able to disclose the data for the time being. If someone would like to request data or has questions about this study, they can contact the corresponding author.
